# Povidone-Iodine Antimicrobial Activity In Vitro Against Periodontal Bacterial Pathogens

**DOI:** 10.7759/cureus.101128

**Published:** 2026-01-08

**Authors:** Thomas E Rams, Chander S Gupta

**Affiliations:** 1 Department of Periodontology and Oral Implantology, Temple University School of Dentistry, Philadelphia, USA

**Keywords:** in vitro, periodontal, periodontitis, povidone-iodine, subgingival microbiota

## Abstract

Background

Povidone-iodine (PV-I) is known to be active in vitro against periodontal bacterial pathogens, but previous studies most often used ≥5-minute contact times for PV-I testing and/or evaluated laboratory reference strains of bacterial species. This study further examined the antimicrobial effects of PV-I by using a 60-second in vitro treatment time for 10% and 5% PV-I on freshly recovered clinical isolates of subgingival biofilm bacteria from severe human periodontitis lesions.

Methods

Subgingival biofilm samples from 22 adults with severe periodontitis were mixed in vitro with 10% PV-I, 5% PV-I, or no PV-I (n = 22 samples per group), with residual PV-I neutralized after 60 seconds with sodium thiosulfate. The samples were then inoculated onto enriched Brucella blood agar (EBBA), with samples not treated with PV-I additionally plated onto EBBA supplemented with breakpoint concentrations of either amoxicillin, clindamycin, doxycycline, or metronidazole to detect antibiotic-resistant test species. After 7 days of anaerobic incubation, total viable microbial counts and selected red/orange complex periodontal pathogens (*Porphyromonas gingivalis*, *Tannerella forsythia*, *Prevotella intermedia/nigrescens*, *Parvimonas micra*, *Campylobacter rectus*, *Fusobacterium nucleatum*, and *Streptococcus constellatus*) were phenotypically identified and quantitated on the EBBA plates, with additional cultivable isolates from PV-I-treated samples identified using matrix-assisted laser desorption-ionization time-of-flight (MALDI-TOF) mass spectrometry.

Results

Subgingival biofilm samples treated in vitro for 60 seconds with 10% or 5% PV-I yielded significantly lower mean total viable microbial counts (60-68% less) and significantly lower mean total cultivable proportions of red/orange complex periodontal pathogens (0.5%-0.7%) than samples not exposed to PV-I (14.8%) (P < 0.001), with no statistically significant differences between 10% and 5% PV-I in vitro treatments. All evaluated red/orange complex periodontal pathogens were culture-negative in 21 (95.5%) and 19 (86.4%) of the 22 subgingival biofilm samples after 10% and 5% PV-I in vitro treatment, respectively. Antibiotic-resistant and antibiotic-susceptible red/orange complex periodontal pathogens were similarly sensitive in vitro to 10% and 5% PV-I. *Streptococcus* species, particularly *Streptococcus oralis*, were the most prevalent cultivable isolates in subgingival samples treated in vitro with 10% or 5% PV-I for 60 seconds.

Conclusions

Both 10% and 5% PV-I significantly suppressed total viable microbial counts and red/orange complex periodontal pathogens, but not periodontal health-associated *Streptococcus* species, during 60 seconds of in vitro treatment on subgingival biofilm samples from patients with severe periodontitis, with no statistically significant differences in the antimicrobial activity of the two PV-I concentrations. These in vitro PV-I susceptibility findings with freshly isolated subgingival biofilm bacteria further support the clinical use of PV-I in periodontal therapy as an adjunct to mechanical root debridement in altering a pathogenic subgingival microbiome toward one compatible with periodontal health.

## Introduction

A primary goal of human periodontitis therapy is to suppress periodontopathic bacteria in subgingival biofilms and re-establish a health-associated microbiome at periodontal sites. Conventional mechanical-surgical periodontal therapy may not adequately alter periodontopathic bacterial populations in some periodontitis patients, predisposing them to further progressive periodontal breakdown [1]. For periodontitis patients harboring a persistent pathogenic subgingival microbiome after periodontal pocket debridement or surgical reduction, adjunctive topical and/or systemic antimicrobial chemotherapy may improve periodontal treatment outcomes [1].

Povidone-iodine (PV-I) is a widely available, low-cost iodophor antiseptic agent composed of iodine combined with a synthetic hydrophilic polymer carrier (polyvinylpyrrolidone) devoid of intrinsic antibacterial activity [2]. However, polyvinylpyrrolidone increases the water solubility and stability of iodine in PV-I and facilitates the slow release of free iodine, which provides the germicidal effects of PV-I, into treated sites [2]. PV-I exerts broad-spectrum microbicidal activity against vegetative bacteria, microbial biofilms, bacterial spores, fungi, mycobacteria, protozoa, and viruses, including herpesviruses [3,4]. Since its clinical introduction in the mid-1950s, PV-I has been widely used to disinfect skin, hands, mucosa, surgical wounds, body cavities, and eye surfaces [3,5]. A negligible-to-low risk of allergic reactions, contact sensitivity, tissue staining, cytotoxicity, thyroid dysfunction, and/or induction of bacterial resistance occurs with short-term clinical use of PV-I, including topical applications in the oral cavity [3,5].

In the treatment of human periodontal disease, PV-I provides favorable clinical results when professionally applied into subgingival sites as an adjunct to mechanical periodontal debridement therapy. A recent systematic review and meta-analysis found that professional in-office irrigation of PV-I into periodontal pockets during mechanical root instrumentation increased gains of clinical periodontal attachment level (CAL) at both 6 months and 12 months post-treatment, and reduced periodontal probing depths at 12 months post-treatment, significantly more than conventional mechanical root debridement alone [6]. In a 13-year clinical trial, 0.1% PV-I used as an ultrasonic scaler coolant during initial periodontal therapy and periodontal maintenance care led to significantly greater gains in CAL in initially deep (≥6 mm) periodontal sites at 1 year post-treatment than water-cooled ultrasonic root debridement [7]. This therapeutic advantage in CAL improvement with PV-I topical chemotherapy at 1 year post-treatment was maintained over the subsequent 12 years. Moreover, less recurrent periodontal breakdown was found in PV-I-treated patients compared to patients treated with water as the ultrasonic scaler coolant (13.4% of patients with further marked CAL loss in the PV-I-treated group versus 25.2% of patients treated with water as the ultrasonic scaler coolant; 1.9-fold difference) [7].

Consistent with these clinical findings, significantly better reductions in total cultivable periodontal pathogen counts in subgingival biofilms were noted at 5 weeks post-treatment when 10% PV-I was continuously irrigated for 5 minutes into deep human periodontal pockets after subgingival root debridement, as compared to mechanical periodontal instrumentation alone (a 7-fold greater percentage of deep periodontal pockets had a >95% reduction in total periodontal pathogen counts with 10% PV-I pocket irrigation) [8].

Despite these favorable clinical and microbiological outcomes, many questions involving the application of PV-I in periodontal therapy remain unresolved, such as which concentration of PV-I is preferred and how long a treatment contact time period is needed between PV-I and targeted pathogenic bacteria in periodontal pockets. Previous in vitro studies on the antimicrobial effects of PV-I on periodontal bacterial pathogens most often employed ≥5-minute treatment contact times [9-11] and/or tested laboratory reference strains of microorganisms [9-14], even though PV-I irrigants mostly wash out of periodontal pockets within 5 minutes of their subgingival delivery [15], and laboratory-adapted reference strains of bacteria frequently develop altered properties and decreased virulence in comparison to freshly recovered wild-type clinical isolates [16].

Since PV-I is markedly reduced within 60 seconds after in vivo placement into subgingival sites [15], the objectives of this study were to use a brief, clinically relevant treatment contact time of 60 seconds in vitro to: (1) evaluate the antimicrobial effects of 10% and 5% PV-I on fresh wild-type clinical isolates of selected red/orange complex periodontal bacterial pathogens isolated from severe human periodontitis lesions; (2) identify additional cultivable isolates surviving in PV-I-treated subgingival samples; and (3) determine whether the antimicrobial effects of PV-I differ between antibiotic-resistant versus antibiotic-susceptible red/orange complex periodontal pathogens.

Parts of this article were previously posted in the Temple University library in 2019 as part of a Master of Science thesis by Gupta CS.

## Materials and methods

Study design and setting

A cross-sectional laboratory study of the in vitro antimicrobial activity of 10% and 5% PV-I against subgingival biofilm bacteria from patients with severe periodontitis was carried out at the Oral Microbiology Testing Service (OMTS) Laboratory (Director: Thomas E. Rams) at the Temple University School of Dentistry, Philadelphia, USA. The OMTS Laboratory was licensed for high-complexity bacteriologic analysis and bacterial susceptibility testing by the Pennsylvania Department of Health and certified by the United States Centers for Medicare and Medicaid Services to be in compliance with Clinical Laboratory Improvement Amendments (CLIA) standards required of clinical laboratories engaged in diagnostic testing of human specimens in the United States. The Temple University Institutional Review Board classified this study as exempt since it involved secondary use of subgingival samples anonymized with removal of unique patient identifiers and no human subject-investigator contact or interaction.

Subgingival biofilm samples

Subgingival biofilm samples from three deep periodontal pockets (≥7 mm) with bleeding on probing in each of 22 adults (9 male, 13 female; mean age 54.3 ± 8.7 years (SD)) with severe periodontitis were obtained by practicing periodontists in the United States, who followed a standardized sampling protocol using sterile paper points. The samples were pooled per patient into single glass sampling vials containing VMGA III transport medium and delivered within 24 hours to the OMTS Laboratory, as previously described [17]. The patient samples were submitted by the periodontists for microbiological testing for periodontal treatment planning purposes and were secondarily used in this study after removal of unique patient identifiers.

PV-I in vitro treatment of subgingival biofilm samples

The 22 subgingival biofilm samples were used to evaluate the antimicrobial effects of 10% and 5% PV-I after a 60-second in vitro treatment contact time using methodology previously employed to assess the in vitro bactericidal effects of silver diamine fluoride and molecular iodine mouthrinses on human subgingival biofilm bacteria [18,19].

At the OMTS Laboratory, the glass sampling vials containing subgingival patient samples were warmed to 37°C for 10 minutes to liquefy gelatin in the VMGA III transport medium and vortexed at the maximal setting for 45 seconds to mechanically disperse sampled microorganisms from the paper points. Serial 10-fold sample dilutions were then prepared with an anaerobic dispersion solution composed of 0.25% tryptose, 0.25% thiotone E peptone, and 0.5% NaCl [17].

For each of the 22 subgingival patient samples, 1.0 mL aliquots of 10^-^⁶ sample dilutions were pipette-mixed (aspiration/expulsion repeated twice, with care taken to avoid introduction of air) in Eppendorf tubes on a laboratory benchtop at room temperature (approximately 22°C) with either no PV-I, 0.05 mL of 10% PV-I (Povidone-Iodine 10% USP, CVS Health, Woonsocket, RI, USA), or 0.05 mL of 5% PV-I (freshly created immediately before use by diluting 0.025 mL of 10% PV-I with 0.025 mL of sterile distilled water), resulting in 22 subgingival samples per group. After a 60-secondin vitro treatment contact time period, residual PV-I in each PV-I-treated mixture was neutralized with 0.05 mL of 3% sodium thiosulfate, which by itself does not affect bacterial viability [19]. Using a sterile bent glass rod, the mixtures were plated in duplicate onto pre-reduced enriched Brucella blood agar (EBBA), composed of 4.3% Brucella agar (BBL Microbiology Systems, Cockeysville, MD, USA) supplemented with 0.3% bacto-agar, 5% defibrinated sheep blood, 0.2% hemolyzed sheep red blood cells, 0.0005% hemin, and 0.00005% menadione [17]. The EBBA-inoculated plates were incubated at 37°C for 7 days in an upright heated incubator (Caron, Marietta, OH, USA) in jars containing an 85% N₂-10% H₂-5% CO₂ anaerobic atmosphere introduced by an Anoxomat™ Mark II automatic jar evacuation-replacement system (Advanced Instruments, Inc., Norwood, MA, USA).

Additional 0.1 mL aliquots of subgingival biofilm samples not treated in vitro with PV-I were plated onto anaerobically incubated EBBA supplemented with breakpoint concentrations of either amoxicillin at 8 mg/L, clindamycin at 4 mg/L, doxycycline at 4 mg/L, or metronidazole at 16 mg/L to detect antibiotic-resistant test species, as previously described [17], which was denoted by test species growth on antibiotic-supplemented EBBA plates.

After incubation, the total number of microbial colony-forming units (CFUs) per EBBA culture plate was quantitated using an automated colony counter system (AccuCount™ 1000 Macroscopic Automated Colony Counter, BioLogics, Inc., Manassas, VA, USA) to determine total viable microbial counts, and established phenotypic criteria were used to presumptively identify and enumerate selected red/orange complex periodontal pathogens, including *Porphyromonas gingivalis*,* Tannerella forsythia*,* Prevotella intermedia/nigrescens*,* Parvimonas micra*,* Campylobacter rectus*,* Fusobacterium nucleatum*,and* Streptococcus constellatus* [17]. The cultivable proportional recovery of red/orange complex periodontal pathogens was calculated as the percent recovery of each species’ CFUs among total viable microbial counts per subgingival patient sample. Red/orange complex species that were culture-negative in PV-I-treated subgingival patient samples, but culture-positive in samples not treated with PV-I, were considered sensitivein vitro to the antimicrobial activity of the tested PV-I concentration within 60 seconds.

Additional cultivable isolates in PV-I-treated subgingival biofilm samples, other than the evaluated red/orange complex periodontal pathogens, were identified using matrix-assisted laser desorption-ionization time-of-flight (MALDI-TOF) mass spectrometry and MALDI Biotyper analytic software (Bruker Daltonics, Billerica, MA, USA), following previously described procedures [20].

**Figure 1:**
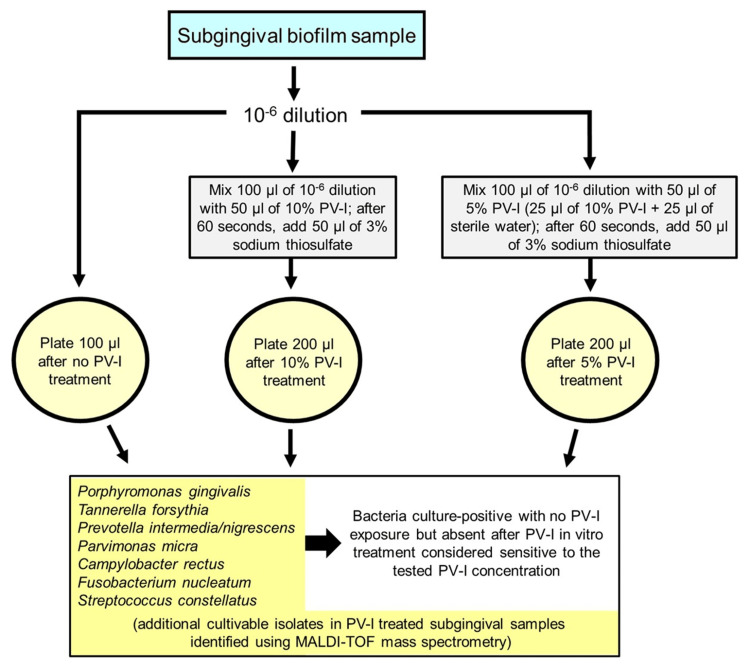
Flow chart of in vitro treatment of subgingival biofilm samples from patients with severe periodontitis with 10% and 5% PV-I. PV-I: Povidone-iodine.

Figure 1 summarizes the study protocol employed for evaluating 10% and 5% PV-I in vitro treatment of subgingival biofilm samples from patients with severe periodontitis.

Data analysis

Total viable microbial counts were tabulated and transformed to mean log₁₀ values with standard error (SE) for study samples treated or not treated in vitro with PV-I. For each red/orange complex test species, the number and proportion of species-positive patient samples were determined, along with the mean subgingival cultivable proportional recovery and SD, as well as the number and proportion of samples with species recovered after 10% or 5% PV-I in vitro treatment. Total subgingival cultivable proportions of red/orange complex periodontal pathogens per patient were calculated by summing individual species data for each patient sample and then averaging the values across all patient samples [18,19].

Paired t-tests compared patient samples treated and not treatedin vitrowith either 10% or 5% PV-I for differences in mean total viable microbial counts and mean total subgingival proportions of red/orange complex periodontal pathogens. For each test antibiotic, two-tailed Fisher’s exact tests compared the distribution of antibiotic-resistant versus antibiotic-susceptible red/orange complex periodontal pathogens per patient in their sensitivity to 10% and 5% PV-I in vitro treatment. A p-value of <0.05 was required for statistical significance in all statistical testing. Cohen’s d was used to estimate the effect size of statistically significant differences, with values ≥0.8 indicating a large effect size. The PC-based STATA/SE 17.0 for Windows (StataCorp PL, College Station, TX, USA) 64-bit statistical software package was used for data analysis.

## Results

PV-I effects on total viable microbial counts

**Figure 2:**
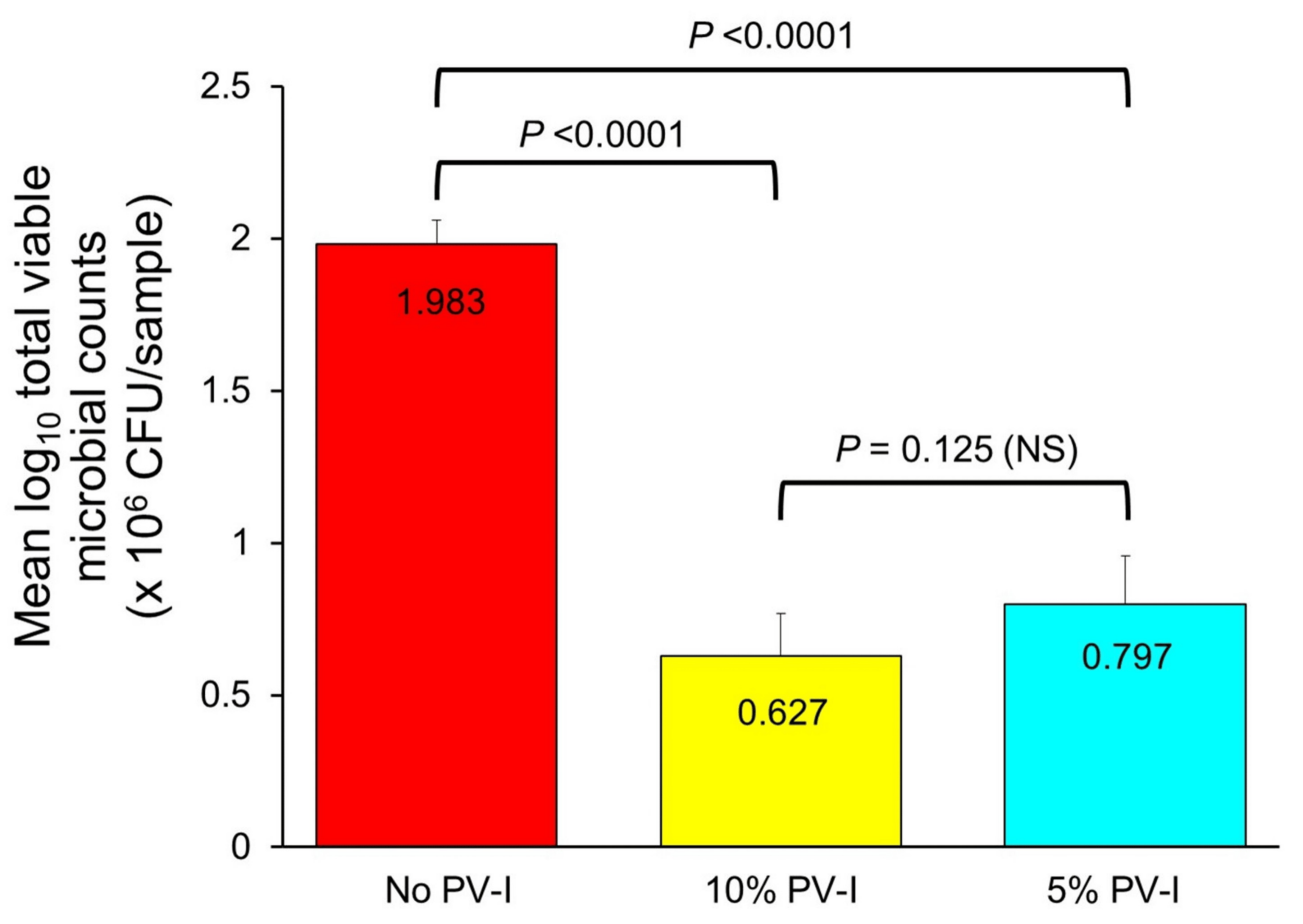
Average total viable microbial counts in subgingival biofilm samples treated and untreated in vitro for 60 seconds with either 10% or 5% PVP-I. Paired t-tests were used to assess mean differences between groups. NS: Not statistically significant; PVP-I: Povidone-Iodine.

Total viable microbial counts averaged 1.983 ± 0.08 (SE) log₁₀ × 10⁶ CFU in subgingival patient samples not exposed to PV-I, 0.627 ± 0.16 log₁₀ × 10⁶ CFU in samples treated in vitro with 10% PV-I, and 0.797 ± 0.14 log₁₀ × 10⁶ CFU in samples treated with 5% PV-I. Total viable microbial counts in subgingival samples treated with either 10% or 5% PV-I were significantly lower than in samples not exposed to PV-I (p values <0.0001, paired t-test; Cohen’s d = 1.7 for 10% PV-I, 1.5 for 5% PV-I), but were not significantly different from each other (p = 0.125, paired t-test) (Figure 2).

PV-I effects on red/orange complex periodontal pathogens

One or more of the evaluated red/orange complex periodontal pathogens were culture-positive in all subgingival samples not treated in vitro with PV-I. Table 1 lists the species isolated and their in vitro resistance to selected antibiotics.

**Table 1:** Cultivable recovery and in vitro antibiotic resistance of red/orange complex periodontal pathogens in untreated (PVP-I-free) subgingival biofilm samples. ND: Not detected; AMOX: Amoxicillin; CLIN: Clindamycin; DOXY: Doxycycline; MET: Metronidazole; PV-I: Povidone-Iodine

Bacterial species	No. (%) of culture-positive patients	% species recovery in culture-positive patients ± SD	Range %	Antibiotic resistance in vitro (No. of patients)
Red complex species				
Porphyromonas gingivalis	4 (18.2)	5.3 ± 7.9	1.1-17.2	ND
Tannerella forsythia	6 (27.3)	2.8 ± 0.6	1.8-3.4	AMOX (1); CLIN (1)
Orange complex species				
Prevotella intermedia/nigrescens	13 (59.1)	4.1 ± 4.6	0.2-14.8	AMOX (4); CLIN (5); DOXY (8)
Parvimonas micra	20 (90.9)	5.5 ± 4.1	0.2-13.8	CLIN (8); DOXY (3)
Campylobacter rectus	9 (40.9)	0.1 ± 0.03	0.1-0.2	ND
Fusobacterium nucleatum	18 (81.8)	6.3 ± 4.3	0.5-15.7	ND
Streptococcus constellatus	1 (4.6)	1.4 ± 0.0	1.4	AMOX (1); CLIN (1); DOXY (1); MET (1)

Orange complex periodontal pathogens were the most prevalent test species recovered. *Parvimonas micra* and *Fusobacterium nucleatum* were the most frequent species, with 20 (90.9%) and 18 (81.8%) of 22 patient samples culture-positive and averaging 5.5% and 6.3% of the total cultivable species, respectively. Among red complex periodontal pathogens, *Tannerella forsythia* averaged 2.8% of subgingival isolates in 6 (27.3%) of 22 patient samples, and *Porphyromonas gingivalis* averaged 5.3% in four species-positive samples (Table 1). Total cultivable proportions of red/orange complex periodontal pathogens per patient averaged 14.8 ± 2.8 (SE) % in subgingival samples not treated with PV-I.

Red/orange complex periodontal pathogens resistant in vitro to either amoxicillin, clindamycin, doxycycline, or metronidazole were detected in 6 (27.3%), 11 (50.0%), 9 (40.9%), and 1 (4.5%) of the 22 patient samples, respectively. A subset of *Prevotella intermedia/nigrescens*, *P. micra*, *T. forsythia*, and* Streptococcus constellatus* were resistant in vitro to amoxicillin and/or clindamycin. Doxycycline-resistant strains included a subset of *P. intermedia/nigrescens*, *P. micra*, and *S. constellatus* isolates, with metronidazole resistance limited to *S. constellatus* in a single patient sample (Table 1).

Table 2 lists cultivable bacterial species in subgingival biofilm samples after PV-Iin vitro treatment.

**Table 2:** Cultivable bacterial species recovered after in vitro PV-I treatment of subgingival biofilm samples. ^a ^Includes (no. of samples): *Streptococcus* spp. (5), *Streptococcus oralis *(4), *Streptococcus vestibularis* (2), *Streptococcus mitis* (1), *Streptococcus salivarius* (1), *Streptococcus anginosus* (1). ^b ^Includes (no. of samples): *Streptococcus oralis* + *Streptococcus intermedius* (1), *Streptococcus* spp. + *Streptococcus intermedius* (1), *Streptococcus salivarius* + *Streptococcus intermedius* (1), *Streptococcus* spp. (5), *Streptococcus oralis* (3), *Streptococcus intermedius* (1), *Streptococcus mitis* (1), *Streptococcus vestibularis* (1), *Streptococcus anginosus* (1). ^c ^Includes (no. of samples): *Staphylococcus* spp. (1), *Staphylococcus aureus* (1). PV-I: Povidone-Iodine.

Bacterial species	No. of subgingival samples culture-positive with species after PV-I in vitrotreatment	
	10% PV-I in vitro treatment	5% PV-I in vitro treatment
Red complex species		
Porphyromonas gingivalis	0	0
Tannerella forsythia	0	0
Orange complex species		
Prevotella intermedia/nigrescens	0	1
Parvimonas micra	0	0
Campylobacter rectus	0	0
Fusobacterium nucleatum	1	2
Streptococcus constellatus	0	0
Additional species		
*Streptococcus *species	14ᵃ	15ᵇ
*Staphylococcus *species	0	2ᶜ
Actinomyces oris	0	1
Capnocytophaga granulosa	0	1
Cutibacterium acnes	0	2

After 10% PV-Iin vitro treatment, all red/orange complex periodontal pathogens were culture-negative in 21 (95.5%) of 22 patient samples. In 13 subgingival samples exhibiting any microbial growth after 10% PV-I in vitro treatment, only various *Streptococcus* species were recovered, with *Streptococcus oralis* most frequently identified to the species level. One additional growth-positive sample had *S. oralis* plus the orange complex species *F. nucleatum* after 10% PV-I in vitro treatment (Table 2).

Following 5% PV-Iin vitro treatment, red/orange complex periodontal pathogens were culture-negative in 19 (86.4%) of 22 samples. Nine subgingival samples with any microbial growth after 5% PV-I in vitro treatment yielded only various *Streptococcus* species. Six other samples had various *Streptococcus *species plus either *P. intermedia/nigrescens*, *F. nucleatum*, *Staphylococcus* species, *Staphylococcus aureus*, *Actinomyces oris*, and/or *Capnocytophaga granulosa*. Two samples showed only *Cutibacterium acnes* (Table 2).

**Figure 3:**
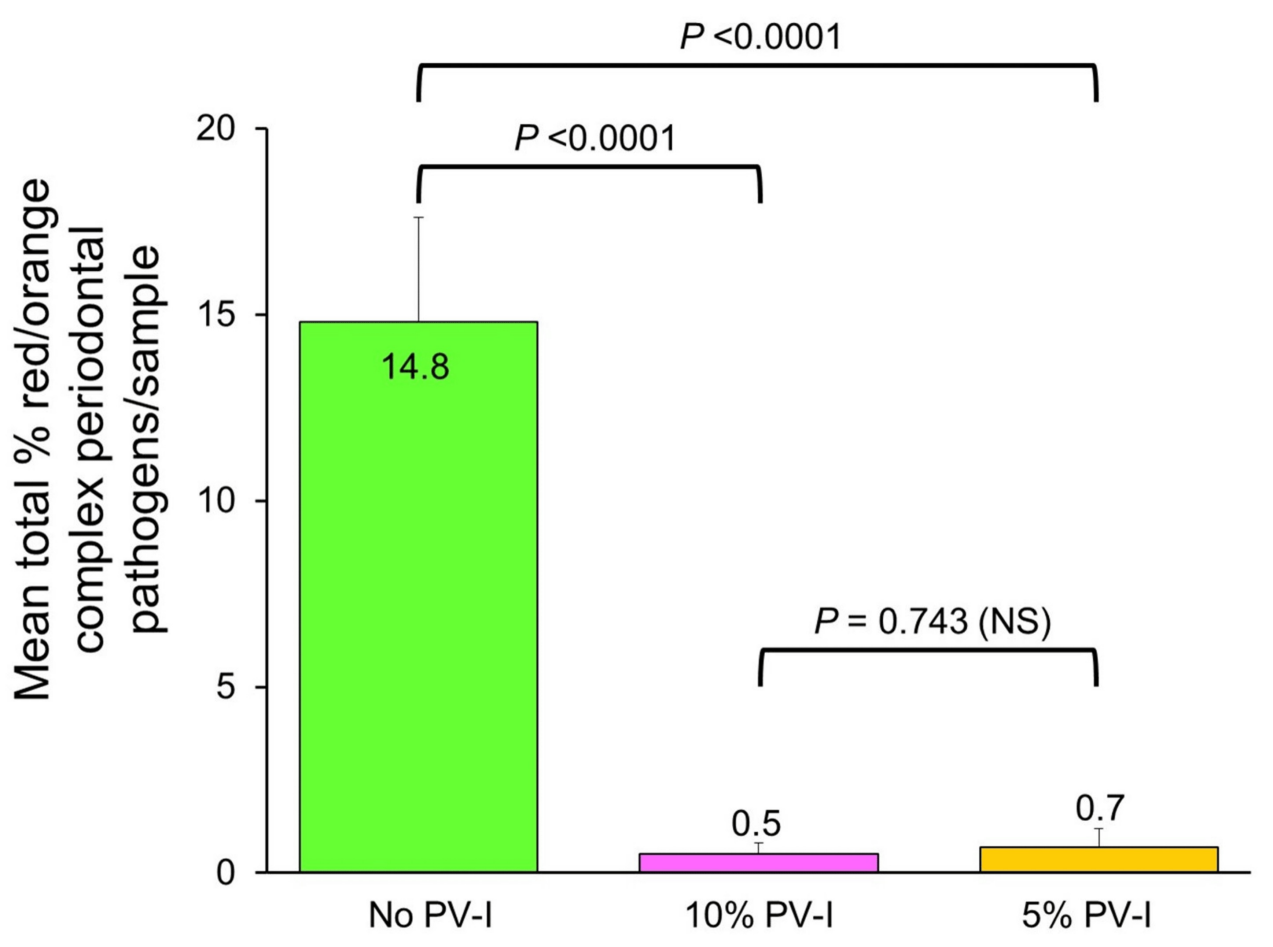
Mean total cultivable proportions of red/orange complex periodontal pathogens in subgingival biofilm samples with or without in vitro treatment with 10% or 5% PV-I. Paired t-tests assessed mean differences between groups. NS: Not statistically significant; PV-I: Povidone-Iodine.

Total cultivable proportions of red/orange complex periodontal pathogens per patient sample averaged 0.5 ± 0.5 (SE) % after 10% PV-I in vitro treatment and 0.7 ± 0.3 % after 5% PV-I in vitro treatment, which were not significantly different from each other (p = 0.743, paired t-test), but were both significantly less than mean levels in subgingival samples not treated with PV-I (p values <0.0001, paired t-tests; Cohen’s d = 1.4 for 10% PV-I, 1.5 for 5% PV-I) (Figure 3).

PV-I effects on antibiotic-resistant versus antibiotic-susceptible red/orange complex species

The antimicrobial effects of 10% and 5% PV-I in vitro treatment on antibiotic-resistant versus antibiotic-susceptible red/orange complex periodontal pathogens are presented in Table 3.

**Table 3:** Antimicrobial effects of 10% and 5% PV-I in vitro treatment on 71 antibiotic-resistant and antibiotic-susceptible isolates of red/orange complex periodontal pathogens. R: antibiotic-resistant red/orange complex periodontal pathogen isolates; S: antibiotic-susceptible red/orange complex periodontal pathogen isolates; NS: no statistically significant difference between antibiotic-resistant and antibiotic-susceptible red/orange complex periodontal pathogen isolates in their sensitivity to PV-I in vitro treatment (two-tailed Fisher’s exact test); PV-I: Povidone-Iodine.

Antibiotic	Susceptibility group	No. of isolates	10% PV-I in vitro treatment: No. (%) rendered culture-negative	p-value	5% PV-I in vitro treatment: No. (%) rendered culture-negative	p-value
Amoxicillin	R	6	6 (100%)		5 (83.3%)	
	S	65	64 (98.5%)	1.000 (NS)	63 (96.9%)	0.236 (NS)
Clindamycin	R	15	15 (100%)		14 (93.3%)	
	S	56	55 (98.2%)	1.000 (NS)	54 (96.4%)	0.515 (NS)
Doxycycline	R	12	12 (100%)		11 (91.7%)	
	S	59	58 (98.3%)	1.000 (NS)	57 (96.6%)	0.431 (NS)
Metronidazole	R	1	1 (100%)		1 (100%)	
	S	70	69 (98.6%)	1.000 (NS)	67 (95.7%)	1.000 (NS)

Among the 71 total red/orange complex periodontal pathogen isolates from 22 subgingival samples tested for in vitro resistance to amoxicillin, clindamycin, doxycycline, and metronidazole, no statistically significant differences were found between antibiotic-resistant and antibiotic-susceptible isolates in each antibiotic test group in their sensitivity to either 10% or 5% PV-I in vitro treatment (Table 3).

All red/orange complex periodontal pathogens resistant in vitro to one or more of the test antibiotics were culture-negative after 10% PV-Iin vitro treatment (Table 3). One *Fusobacterium nucleatum* isolate recovered after 10% PV-I in vitro treatment was not resistant to any of the four test antibiotics. In subgingival samples treated in vitro with 5% PV-I, a recovered *Prevotella intermedia/nigrescens* isolate was resistant to doxycycline, amoxicillin, and clindamycin, whereas two *F. nucleatum* isolates were not resistant to any of the test antibiotics.

## Discussion

The major finding from this study is that 10% and 5% PV-I exerted marked antimicrobial effects against total viable microbial counts and red/orange complex periodontal pathogens in freshly sampled subgingival biofilms from patients with severe periodontitis after only a brief (60-second), clinically relevant in vitro treatment contact time, with no statistically significant differences between 10% and 5% PV-I in their antimicrobial activity. This is in agreement with prior in vitro studies of PV-I on periodontopathic bacteria [9-14] and consistent with in vivo effects of 10% PV-I irrigation into deep human periodontal pockets [8]. However, prior studies either did not test PV-I on fresh wild-type clinical isolates from severe periodontitis lesions or did not employ a brief PV-I in vitro treatment contact time of 60 seconds [9-14], as were both done in the present study.

Importantly, antibiotic-resistant and antibiotic-susceptible red/orange complex periodontal pathogens were found to be similarly sensitive to in vitro treatment with 10% and 5% PV-I (Table 3), a finding not previously reported for periodontopathic bacteria. This is consistent with observations of no antibiotic cross-resistance with PV-I among non-periodontal bacteria [3]. In light of the increasing prevalence of antibiotic-resistant periodontal pathogens in United States periodontitis patients and elsewhere [17,21,22], PV-I applications in the oral cavity may serve as an alternative to antibiotic therapies in combating periodontal infections, particularly those involving antibiotic-resistant species. The combined antimicrobial effects of topical PV-I plus mechanical periodontal debridement [1,8] may also help reduce potential dissemination of antibiotic resistance genes between oral microorganisms, as well as to human microbiome species at other body sites [23].

The finding of gram-positive *Streptococcus* species of low periodontopathic potential (Table 2) [24] as the predominant bacteria surviving after 60 seconds of 10% or 5% PV-I in vitro treatment of subgingival biofilm samples is also unique to the present study. *Streptococcus oralis*, the most prevalent streptococcal species identified to the species level, has a statistically significant inverse relationship with progressive periodontal breakdown (OR = 0.77) and is prominent in the subgingival microbiome of clinically stable and healthy periodontal sites [24]. While PV-I acts against multiple cellular targets to prevent development of PV-I microbial resistance [3], a longer treatment contact time may be needed for PV-I to kill gram-positive cocci, as previously reported with *Streptococcus sanguinis* [25]. With more pronounced bactericidal effects against periodontal bacterial pathogens than *Streptococcus* species after a 60-second PV-I in vitro treatment (Table 2), PV-I in vivo irrigation may potentially give an ecologic advantage to periodontal health-associated microbial species in the post-treatment recolonization of periodontal pockets.

A 60-second PV-I in vitro treatment time was used in the present study to mimic the peak contact time of PV-I solutions placed into inflamed periodontal pockets prior to their progressive clearance by an increased flow of gingival crevicular fluid and/or gingival bleeding [15]. A rapid bactericidal effect is particularly important for antiseptic agents lacking substantivity for oral tissues, such as PV-I, after their non-sustained delivery into periodontal pockets [26]. Rapid clearance of solutions and gels from periodontal pockets limits the efficacy of non-tooth or non-tissue-binding antimicrobial agents that are locally applied in periodontitis treatment and lack rapid microbicidal activity [26]. Clinically, PV-I may be introduced as a solution or gel into subgingival sites via irrigation syringes equipped with narrow-diameter blunt cannulas or applied as an aqueous coolant solution through scaler tips during ultrasonic scaler pocket debridement [1,7,8,15,26]. High-speed evacuation and saliva ejectors should be used by dental professionals to limit patient ingestion or aspiration of PV-I during topical intraoral PV-I therapy [1]. A 5% PV-I concentration or less may also be selected instead of 10% PV-I to further reduce patient iodine exposure. PV-I intraoral use should be avoided in patients hypersensitive to PV-I, with thyroid disease, who are pregnant, nursing, or receiving radioactive iodine therapy [5]. Because subgingival root instrumentation of periodontitis lesions causes gingival tissue bleeding, which reduces the microbicidal activity of PV-I [2] and washes it rapidly out of periodontal pockets [15], a likely best practice is to initially introduce PV-I into periodontal pockets prior to, and replenish it during and after, mechanical periodontal therapy to maximize its antimicrobial effects.

In comparison to PV-I [6-8], several other antiseptic agents suggested for topical periodontal pocket delivery have less documentation of clinical benefit. Chlorhexidine irrigation into periodontal pockets during mechanical root debridement therapy generally provides no adjunctive clinical benefits with respect to improved CAL values [26]. This is likely due to chlorhexidine requiring 5-minute bactericidal concentrations against periodontal pathogens (0.5% to 2%) that exceed levels of chlorhexidine present in commercial products marketed for oral use (0.12% to 0.2%) [9,26]. Moreover, chlorhexidine may be inactivated in periodontal pockets by binding to high levels of serum proteins in gingival crevicular fluid [26]. A similar lack of adjunctive clinical benefit to periodontal debridement therapy occurs with subgingival irrigation of 3% hydrogen peroxide [26], which may be rapidly decomposed by bacterial catalase enzymes in the oral cavity. Interestingly, 3% hydrogen peroxide may potentiate the bactericidal effects of 10% PV-I against various periodontal pathogens in vitro when the two antiseptics are freshly mixed into a 50%-50% aqueous solution [10]. Silver diamine fluoride, which may be applied into subgingival sites via a microbrush applicator, has marked bactericidal activity in vitro against red/orange complex periodontal pathogens [18], but is untested clinically as an adjunct to conventional periodontal treatment procedures. Freshly mixed dilute (0.1% to 0.5%) sodium hypochlorite (household bleach), which is highly effective in patient home oral hygiene procedures as a rinse or oral irrigator solution [1], may also be introduced into periodontal pockets during professionally administered periodontal therapy, but outcome data are limited to date.

Dilution of commercial 10% PV-I products with water, as was performed in the present study to obtain 5% PV-I, may release increased levels of free iodine from the polyvinylpyrrolidone carrier [27]. However, such diluted PV-I solutions may not remain chemically stable or maintain their biocidal potency [28]. This may account for the slightly inferior, but not significantly so, antimicrobial activity of 5% PV-I obtained by dilution with water as compared to undiluted 10% PV-I (Figures 2-3, Table 2). As an alternative, mouthrinses with high concentrations of free iodine are commercially available [19], but are more costly than PV-I and lack clinical trial data evaluating their potential efficacy in periodontal therapy. Both 10% and 5% PV-I in the present study provided similar antimicrobial effectsin vitro against periodontopathic bacteria as were found with two commercial molecular iodine mouthrinses [19], despite the presence of markedly higher levels of free iodine in the mouthrinses. This may be due to the hydrophilic polyvinylpyrrolidone carrier complexed with iodine in PV-I formulations, which, due to its affinity for microbial cell membranes, facilitates delivery and release of free iodine directly to bacterial cell surfaces to initiate protein denaturation, disruption of microbial metabolic pathways, and oxidation of nucleic acid structures leading to cell death [2]. Molecular iodine mouthrinses without polyvinylpyrrolidone complexed to the iodine may be less effective than PV-I in concentrating free iodine at the cell wall of target microorganisms, similar to findings with Lugol's iodine solution [2].

The present study has several limitations. Only in vitro testing of PV-I antimicrobial activity was performed, and it is not known if similar PV-I antimicrobial effects occur in vivo within periodontal pockets. The present study data, in which the antimicrobial effects of PV-I were assessed in vitro at room temperature, likely underestimate the antimicrobial activity of PV-I irrigated in vivo into inflamed periodontal pockets with elevated subgingival temperatures [29], since warmer human body temperatures induce release of greater amounts of free iodine from PV-I complexes than cooler room temperatures, markedly increasing its potential germicidal efficiency [30]. Free iodine levels in the PV-I test solutions were not measured, and no testing was performed of PV-I concentrations more dilute than 5%. Only selected cultivable red/orange complex periodontal pathogens were evaluated, without testing of additional periodontal pathogens likely present in the subgingival biofilm samples.

## Conclusions

Both 10% and 5% PV-I significantly suppressed total viable microbial counts and red/orange complex periodontal pathogens in severe periodontitis biofilm samples after 60 seconds of in vitro treatment, with no statistically significant differences in the antimicrobial activity of 10% and 5% PV-I. Antibiotic-resistant and antibiotic-susceptible red/orange complex periodontal pathogens were similarly sensitive in vitro to 10% and 5% PV-I. Periodontal health-associated *Streptococcus* species were the most prevalent cultivable isolates after subgingival samples were treated in vitro with 10% or 5% PV-I for 60 seconds. These PV-I in vitro susceptibility findings with freshly isolated subgingival biofilm bacteria further support the clinical use of PV-I in periodontal therapy as an adjunct to mechanical root debridement in altering a pathogenic subgingival microbiome toward one compatible with periodontal health.
